# Zhibitai and low-dose atorvastatin reduce blood lipids and inflammation in patients with coronary artery disease

**DOI:** 10.1097/MD.0000000000006104

**Published:** 2017-02-17

**Authors:** Yuhong Zhao, Ran Peng, Wang Zhao, Qiong Liu, Yuan Guo, Shuiping Zhao, Danyan Xu

**Affiliations:** a Internal Cardiovascular Medicine, Second Xiangya Hospital of Central South University, Changsha, Hunan; b Affiliated Peace Changzhi Medical College Hospital, Changzhi, Shanxi, China.

**Keywords:** atorvastatin, cardiotrophin-1, cholesterol, high sensitive-C reactive protein, zhibitai

## Abstract

**Background:**

Atorvastatin decreases blood lipids but is associated with side effects. Zhibitai is a traditional Chinese medicine used to treat blood lipid disorders. The objective of this study is to evaluate the lipid-lowering effect, antiinflammatory effect, and adverse events of zhibitai combined to atorvastatin in patients with coronary heart diseases (CHDs).

**Methods:**

Patients with CHD (n = 150) were randomized to: zhibitai 480 mg + atorvastatin 10 mg (ZA10 group), atorvastatin 20 mg (A20 group), and atorvastatin 40 mg (A40 group). Lipid profile, cardiotrophin-1 (CT-1), and C-reactive protein (CRP) were measured after 4 and 8 weeks of treatment. Self-reported side effects, liver function, kidney function, and creatine kinase levels were monitored.

**Results:**

After 8 weeks, triglycerides, total cholesterol (TC), LDL-cholesterol (LDL-C), and apolipoprotein B_100_ (ApoB_100_) levels were decreased in the ZA10 group (−64%, −37%, −46%, and −54%, respectively, compared with baseline), and these changes were similar to those of the A40 group (*P* > 0.05). CT-1 and high sensitivity-C reactive protein (hs-CRP) levels were significantly decreased in the ZA10 group after 4 and 8 weeks (4 weeks: −73% and 96%; 8 weeks: −89% and −98%; all *P* *<* 0.01), without differences among the 3 groups (*P* > 0.05). After 8 weeks of treatment, adverse events (abdominal distention, nausea, vomiting, and hunger) were found in 4, 5, and 7 patients in the ZA10, A20, and A40 groups, respectively.

**Conclusion:**

ZA10 significantly reduced triglycerides, TC, LDL-C, ApoB, CT-1, and hs-CRP levels in patients with CHD, similar to the effects of A40 and A20, but ZA10 lead to fewer adverse events.

## 1 Introduction

Health is the most important construct of our life. The 21st century enabled us to live in improved living conditions. However, although our health status changed, many chronic stages of diseases were integrated into our lives.^[1]^ Physical inactivity is one of the risk factors of atherosclerosis and obesity,^[2]^ and may even be the most important. There is now overwhelming evidence that regular physical activity has important and wide-ranging health benefits. These range from reduced risk of chronic diseases such as heart disease, type 2 diabetes, and some cancers to enhance function and preservation of function with age.^[3]^ Existing research shows that proper physical exercise, a healthy environment and balanced nutrition,^[4]^ and good state of mind is an effective factor for the prevention of coronary heart disease (CHD).

Atherosclerosis leads to the narrowing of the lumen of coronary arteries. Eventually, progressive plaque thickening and/or rupture may lead to angina and/or myocardial infarction (MI).^[5–8]^ In the United States, the prevalence of CHD is about 6.2% in people ≥20 years old.^[9]^ The rates of major cardiovascular events are higher in developing countries compared with developed ones.^[10]^ Mortality from ischemic heart disease is the leading cause of mortality worldwide, with 12.7% of the total mortality in 2008.^[11]^ Hyperlipidemia is a major risk factor for the development and progression of atherosclerosis.^[5–8]^

Statins are 3-hydroxy-3-methyl-glutaryl-coenzyme A reductase inhibitors; this enzyme normally catalyzes the rate-limiting step of cholesterol synthesis.^[12]^ Therefore, statins induce cholesterol depletion within the hepatocytes, leading to the upregulation of the low-density lipoprotein (LDL) receptor in order to obtain cholesterol from blood LDL particles.^[13]^ Statins also decrease C-reactive protein (CRP) levels, but their effect on inflammation is not consistent.^[14]^ Statins are the drugs of choice for patients at risk of CHD or CHD progression.^[15]^ Statins significantly reduce the incidence of all-cause mortality and major coronary events as compared to control in both secondary and primary prevention. Atorvastatin was significantly more effective than pravastatin (OR 0.65, 95% CI 0.43–0.99) and simvastatin (OR 0.68, 95% CI 0.38–0.98) for secondary prevention of major coronary events.^[16]^ Atorvastatin is a 3rd-generation statin with a good efficacy and adequate safety profile.^[17]^

Nevertheless, statins are associated with adverse effects such as myopathy, hepatic toxicity, hyperglycemia, and impaired steroid production; rhabdomyolysis and death are also possible.^[18]^ The risk of adverse effects increases with the higher doses.^[19]^ Statins have been shown to reduce the risk of all-cause mortality among individuals with clinical history of CHD. However, it remains uncertain whether statins have similar mortality benefit in a high-risk primary prevention setting. One literature-based meta-analysis did not find evidence for the benefit of statin therapy on all-cause mortality in a high-risk primary prevention set-up.^[20]^ The findings suggest that intensive dose atorvastatin therapy does not attenuate the progression of coronary calcification compared with standard dose atorvastatin therapy over 12 months.^[21]^

Chinese medicine has been regarded as a kind of complementary therapies in the Western countries. However, it has been one of the mainstream therapies in some Asian countries, such as Taiwan, Korea, and China, including acupuncture, moxibustion, Chinese orthopedics and traumatology, and Chinese herbal medicine (CHM).^[22]^ CHM has a long history and is widely used in the prevention and treatment of a variety of diseases worldwide. CHM has been used to treat stroke for thousands of years. The findings suggest that bioactive components of CHM may improve neurological function, reduce infarct volume, and promote endogenous neurogenesis, including proliferation, migration, and differentiation of neural stem cells after ischemic stroke.^[23]^ Zhibitai is a traditional Chinese medicine that is made of naturally occurring statins and it is used to treat blood lipid disorders. Previous studies have shown that zhibitai was comparable to atorvastatin in terms of lipid-lowering and antiinflammatory effects.^[24–26]^ Therefore, zhibitai could be an appropriate alternative to chemical lipid-lowering drugs.

Therefore, the aim of the present study was to further evaluate the lipid-lowering effect, antiinflammatory effect, and adverse events of zhibitai combined to atorvastatin 10 mg in patients with CHDs, and to investigate the advantages of the combination compared with higher doses of atorvastatin.

## 2 Methods

### 2.1 Patients

This was a prospective study of patients with CHD treated at the Inpatient and Outpatient Departments of the Second Xiangya Hospital of Central South University and at the Peace Hospital Affiliated to Changzhi Medical College (Shanxi, China) between October 2010 and May 2011. The study was approved by the ethical committees of the 2 participating hospitals. All patients signed an informed consent form.

The inclusion criteria were: diagnosis of CHD with signs and symptoms of MI (typical chest pains and pain-related ST-T changes; typical chest pains and positive treadmill exercise test; or typical chest pains and coronary angiography showing one or multiple coronary branches with stenosis >50%); >20 but <75 years of age; and volunteered to participate and signed the informed consent form.

The exclusion criteria were: history of MI during the past 3 months; history of cerebrovascular accidents during the past 6 months; history of severe trauma or major surgery; nephrotic syndrome; hypothyroidism; any liver or gallbladder disorders; familial hypercholesterolemia; or secondary hyperlipidemia.

The following drugs were allowed: angiotensin-converting enzyme inhibitor, angiotensin II receptor blockers, calcium channel blockers, clopidogrel, bayaspirin, and β-receptor blockers, as well as other antiplatelet, anticoagulation, antihypertensive, hypoglycemic, and coronary artery dilation drugs such as syncon.

### 2.2 Study design

This was a randomized, parallel study. Patients were randomized using a random number table and sealed envelopes prepared by a statistician. Patients in the zhibitai 480 mg + atorvastatin 10 mg (ZA10) group received zhibitai (480 mg, twice daily, batch #WS_3_–119(Z-119)-2004(Z), Chengdu Di’ao Pharmaceutical Group Corporation, China) and atorvastatin (10 mg, once daily Pfizer Pharmaceutical Co., Ltd.) at night for 8 weeks. The patients in the atorvastatin 20 mg (A20) group received 20 mg of atorvastatin once daily at night for 8 weeks. The patients in the atorvastatin 40 mg (A40) group received 40 mg of atorvastatin once daily at night for 8 weeks.

### 2.3 Testing

Fasting peripheral venous blood was collected in the morning before treatment and after 4 and 8 weeks of treatment. Levels of cardiotrophin-1 (CT-1) (Xinqidi Biological technology Co., Ltd., Wuhan, China), high sensitivity-C reactive protein (hs-CRP, P800, Roche Diagnostics, Germany), triglycerides, total cholesterol (TC), LDL-cholesterol (LDL-C), high-density lipoprotein cholesterol, apoprotein B_100_ (ApoB_100_), apo A_1_, creatine kinase, and markers of liver and renal functions were detected using DXI800 and AU5800 automatic biochemical analyzers (Beckman, United States of America). Twelve-lead ECG was performed routinely.

### 2.4 Statistical analyses

Based on previous studies of zhibitai,^[24–26]^ it was determined that a sample size of 35 per group was necessary to detect a difference at a power of 80% and α = 0.05. SPSS 16.0 (IBM, Armonk, NY) was used for statistical analysis. Continuous data were presented as means ± standard deviation and analyzed using independent *t-*tests for comparisons between 2 groups or paired *t-*tests for comparisons before/after treatment. One-way analysis of variance was used for comparison among the 3 groups, with the Tukey post hoc test. Categorical data were presented using frequencies and percentages and analyzed using the chi-square test. All analyses were two-sided and *P*-values <0.05 were considered statistically significant.

## 3 Results

### 3.1 Characteristics of the patients

A total of 150 patients were randomized (n = 50/group). 1 patient in the ZA10 group was lost to follow-up and 1 patient was excluded because of poor compliance. 2 and 3 patients in the A20 and A40 groups, respectively, were excluded because of poor compliance. Therefore, 48, 48, and 47 patients in the ZA10, A20, and A40 groups, respectively, were included in for the statistical analysis.

There were no significant differences in age, sex, body mass index, smoking history, liver function, renal function, blood lipids, creatine kinase, blood pressure, diabetes, family history of CHD, and drugs among the 3 groups at baseline (all *P* > 0.05) (Table 1).

**Table 1:**
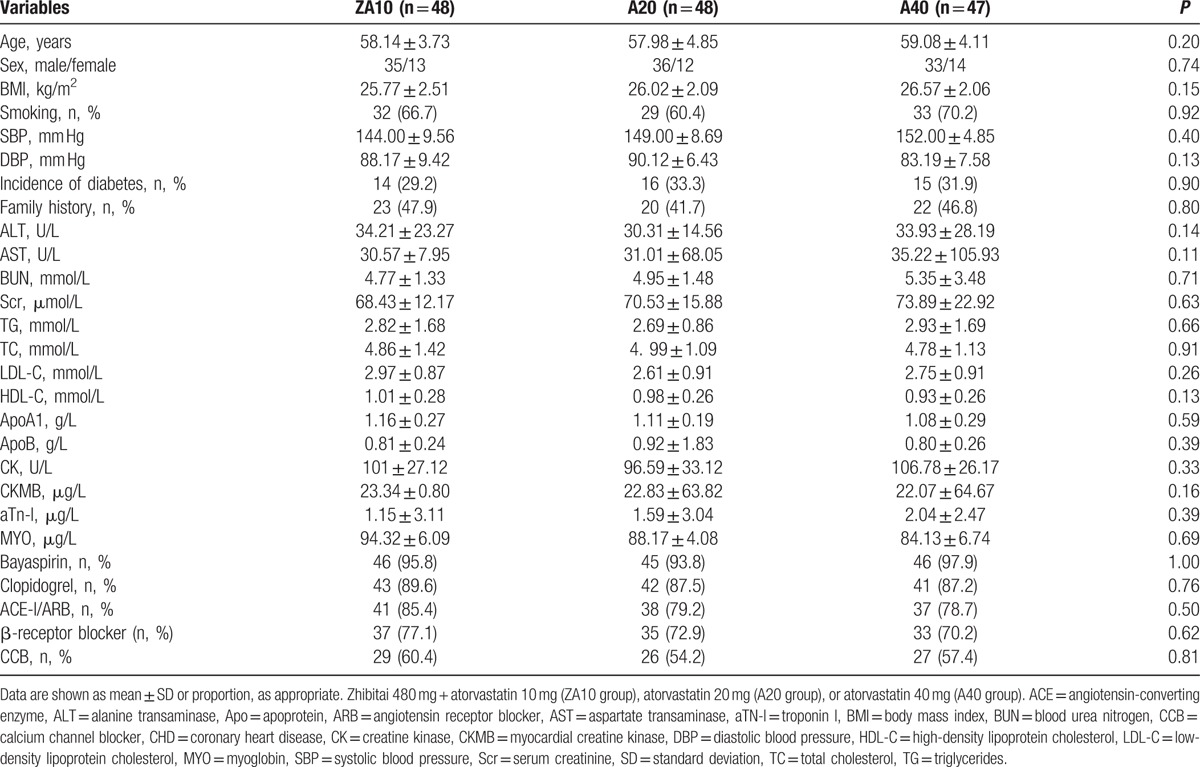
Characteristics of the CHD patients.

### 3.2 Changes in lipid profile after treatment

Compared with baseline, blood lipids after 4 and 8 weeks of treatment were significantly changed in all 3 groups. TC, LDL-C, and ApoB_100_ were decreased significantly compared with baseline (*P* < 0.01), and the resulting levels were similar among all 3 groups (*P* > 0.05 among groups at 4 and 8 weeks). The decreases in TG levels were significant in the ZA10 and A40 groups at 4 and 8 weeks (*P* < 0.01), while the decrease in TG in the A20 group was only significant at 8 weeks (*P* < 0.01). There were no differences in TG levels among the 3 groups at 8 weeks (*P* > 0.05) (Table 2).

**Table 2:**
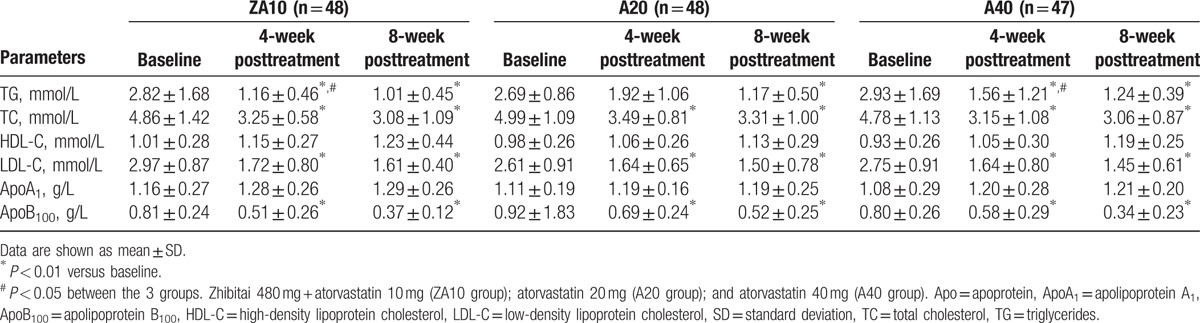
Changes in lipid profile after zhibitai + atorvastatin or atorvastatin treatment.

### 3.3 Changes in CT-1 and hs-CRP after treatment

CT-1 levels were decreased significantly after 4 and 8 weeks of treatment in all 3 groups compared with baseline (*P* < 0.01), and the resulting levels were similar among all 3 groups (*P* > 0.05). hs-CRP levels were also decreased significantly after 4 and 8 weeks in the 3 groups compared with baseline (*P* < 0.05), without difference among the 3 groups (*P* > 0.05) (Table 3).

**Table 3:**
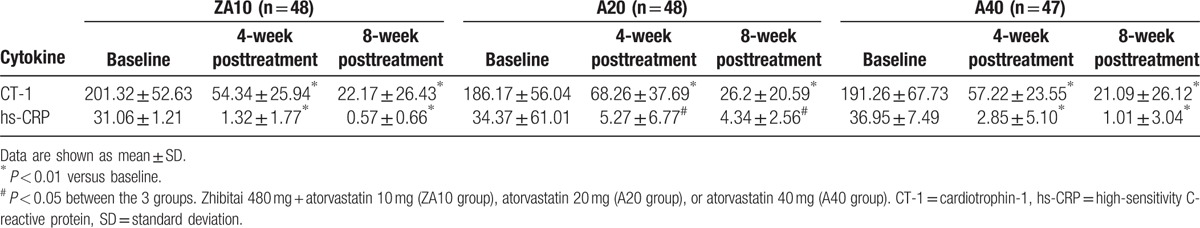
Changes in CT-1 and hs-CRP levels after zhibitai + atorvastatin or atorvastatin treatment.

### 3.4 Liver and renal functions, creatine kinase, and adverse reactions

After 8 weeks of treatment, adverse events (abdominal distention, nausea, vomiting, and hunger) were reported in 4, 5, and 7 patients in the ZA10, A20, and A40 groups, respectively. Before treatment, and after 4 weeks, 8 weeks treatment, incidence of creatine kinase increasing, the incidence of myopathy, the incidence of gastrointestinal adverse reactions have no significant difference in the three groups (all *P* > 0.05), and between the each 2 groups, no significant difference can be observed (*P* > 0.05), There was no significant difference in CK between the three groups before and after treatment (Table 4).

**Table 4:**
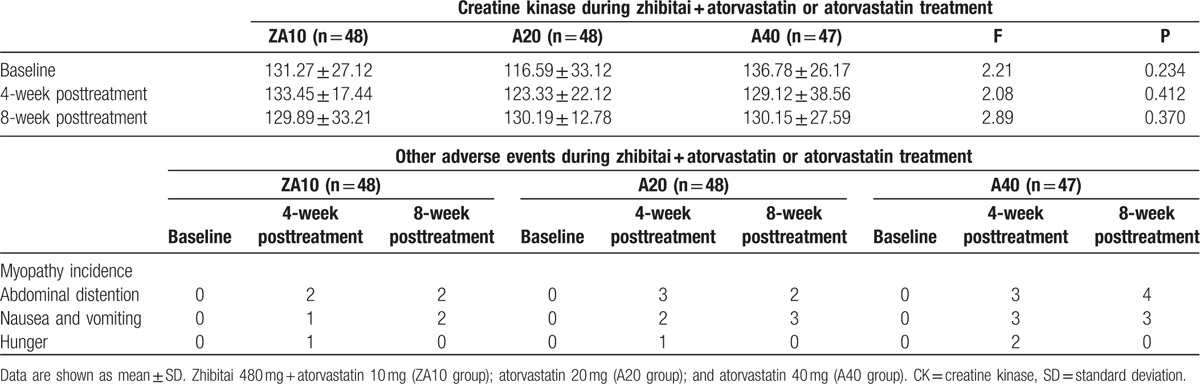
Adverse events during zhibitai + atorvastatin or atorvastatin treatment.

## 4 Discussion

Abnormal lipid metabolism is a major cause and an independent risk factor of CHD. Therefore, lipid-lowering therapy is crucial to the treatment and prevention of CHD.^[27,28]^ Statins are the main currently accepted lipid-lowering treatment.^[13–15]^ However, most intensive lipid-lowering studies tried to achieve better clinical efficacy by increasing the doses of statins, which led to increased incidence of adverse effects.^[19]^

Therefore, the aim of the present study was to evaluate the lipid-lowering effect, antiinflammatory effect, and adverse events of zhibitai combined to atorvastatin 10 mg in patients with CHD. The results showed that TG, TC, LDL-C, and ApoB_100_ levels were decreased in the ZA10 group to a similar extent than in the A40 group. The levels of CT-1 and hs-CRP were significantly decreased in the ZA10 group after 4 and 8 weeks, without difference among the 3 groups. The incidence of adverse events (abdominal distention, nausea, vomiting, and hunger) in the ZA10 group was lower compared with the A20 and A40 groups.

Previous studies have shown that zhibitai used alone could lower blood lipids and inflammatory factors (including hs-CRP and CT-1).^[24–26]^ The present study showed that the combined use of zhibitai and atorvastatin 10 mg could achieve a clinical efficacy comparable to that of higher doses of atorvastatin, without additional adverse effects. In the present study, the extent of TG lowering was higher than that of TC lowering when using zhibitai. As the majority of Asian patients with hyperlipidemia are mainly with increased TG,^[29]^ zhibitai could be more appropriate for patients with mixed hyperlipidemia characterized by increased TG. In addition, zhibitai combined to atorvastatin could achieve even better lipid-lowering effects. Therefore, technically, zhibitai could be suitable for Chinese patients with hyperlipidemia.

Recent studies have shown that increased serum hs-CRP levels are closely associated with CHD and can be used to evaluate the patients’ condition and stability.^[30–32]^ CT-1 is a member of the IL-6 family.^[33]^ Many recent studies have demonstrated that CT-1 is closely associated with the development and progression of CHD.^[34]^ Increased CT-1 levels are found in patients with CHD and are associated with disease severity. The present study showed that the combined use of zhibitai and atorvastatin significantly reduced CT-1 and hs-CRP levels in patients with CHD.

In the present study, there were no significant increases in CK, myopathy, and distinct liver and renal damage in the ZA10 group compared with the A20 or A40 groups, but the number of events was low, preventing any reliable statistical analyses. Nevertheless, previous studies of zhibitai showed a low occurrence of adverse effects.^[24–26]^

The present study is not without limitations. Indeed, the sample size was small and from only 2 hospitals. There were no differences among the groups at 8 weeks, and a noninferiority trial should be performed to confirm this equivalence. Further multicenter prospective trials are necessary to reach firm conclusions about the value of zhibitai for the treatment of hyperlipidemia.

In conclusion, ZA10 in patients with CHD significantly reduced TG, TC, LDL-C, and ApoB levels, as well as the inflammatory cytokines CT-1 and hs-CRP. These changes were similar to those achieved using A40 and A20, but ZA10 led to fewer adverse events.

## 5 Limitations

As the number of cases in this study is small, the observation time is not long enough, so the data and results are one-sided. This will be improved in future research work. The study is only a 1st sign of possible positive influence of herbal therapy, and further studies in the future are necessary to show positive effects on mortality and cardiac events like myocardial infarction or cardiac sudden death. This is the focus of our study work in the future.

## Acknowledgments

The authors thank National Natural Science Foundation of China (No. 81672264, 81372117) for the support.
